# Expression of *Aspergillus nidulans phy* Gene in *Nicotiana benthamiana* Produces Active Phytase with Broad Specificities

**DOI:** 10.3390/ijms150915571

**Published:** 2014-09-03

**Authors:** Tae-Kyun Oh, Sung Oh, Seongdae Kim, Jae Sung Park, Nagarajan Vinod, Kyung Min Jang, Sei Chang Kim, Chang Won Choi, Suk-Min Ko, Dong Kee Jeong, Rajangam Udayakumar

**Affiliations:** 1 Department of Biology & Medicinal Science, Pai Chai University, Daejeon 302-735, Korea; E-Mails: ohtk0@pcu.ac.kr (T.-K.O.); 5star@pcu.ac.kr (S.O.); khboy111@pcu.ac.kr (S.K.); parkjs@kgicenter.com (J.S.P.); biovinz@yahoo.com (N.V.); cardgame@hanafos.com (K.M.J.); kimsc@pcu.ac.kr (S.C.K.); 2 Research Institute for Subtropical Agriculture and Animal Biotechnology, Jeju National University, Jeju-si 690-756, Jeju Special Self-Governing Province, Korea; E-Mail: heyna@jejunu.ac.kr; 3 Faculty of Biotechnology, Jeju National University, Jeju-si 690-756, Jeju Special Self-Governing Province, Korea; E-Mail: dkjeong@jejunu.ac.kr; 4 Department of Biochemistry, Government Arts College (Autonomous), Kumbakonam-612 001, Tamilnadu, India; E-Mail: udayabiochem@yahoo.co.in

**Keywords:** *Aspergillus nidulans*, *phy* gene, *Escherichia coli*, polyclonal antibody, transient expression, *Agrobacterium* infiltration, oral administration

## Abstract

A full-length phytase gene (*phy*) of *Aspergillus nidulans* was amplified from the cDNA library by polymerase chain reaction (PCR), and it was introduced into a bacterial expression vector, pET-28a. The recombinant protein (rPhy-E, 56 kDa) was overexpressed in the insoluble fraction of *Escherichia coli* culture, purified by Ni-NTA resin under denaturing conditions and injected into rats as an immunogen. To express *A. nidulans* phytase in a plant, the full-length of *phy* was cloned into a plant expression binary vector, pPZP212. The resultant construct was tested for its transient expression by *Agrobacterium*-infiltration into *Nicotiana benthamiana* leaves. Compared with a control, the agro-infiltrated leaf tissues showed the presence of *phy* mRNA and its high expression level in *N.* *benthamiana.* The recombinant phytase (rPhy-P, 62 kDa) was strongly reacted with the polyclonal antibody against the nonglycosylated rPhy-E. The rPhy-P showed glycosylation, two pH optima (pH 4.5 and pH 5.5), an optimum temperature at 45~55 °C, thermostability and broad substrate specificities. After deglycosylation by peptide-*N*-glycosidase F (PNGase-F), the rPhy-P significantly lost the phytase activity and retained 1/9 of the original activity after 10 min of incubation at 45 °C. Therefore, the deglycosylation caused a significant reduction in enzyme thermostability. In animal experiments, oral administration of the rPhy-P at 1500 U/kg body weight/day for seven days caused a significant reduction of phosphorus excretion by 16% in rat feces. Besides, the rPhy-P did not result in any toxicological changes and clinical signs.

## 1. Introduction

Phytase (InsP6 phosphohydrolase) catalyzes the sequential hydrolysis of phytic acid to produce less phosphorylated myo-inositol derivatives and inorganic phosphorus (P). Monogastric animals produce little intestinal phytase activity, resulting in the excretion of undigested phytate-P into the environment. Animal feeds supplemented with phytase increase the bioavailability of phytic acid-bound phosphate and prevent phosphate pollution in the environment. A number of microbial phytases have been isolated from bacteria, yeast and fungi [1,2]. Among these, fungal phytases are well characterized [3,4,5,6,7,8,9,10,11]. Especially, *Aspergillus* *niger* is known to produce two other extracellular acid phosphatases, pH 2.5 optimum acid phosphatase (PhyB, EC 3.1.3.2) and pH 5.0–6.0 optimum acid phosphatase (PhyA, EC 3.1.3.8) [7,8]. PhyA, a member of the subfamily histidine acid phosphatase (HAP), efficiently degrades phytic acid in an acidic pH range [9,12,13], and it is commercialized under the name of Natuphos, used as an animal feed additive. PhyB also belongs to HAP [13], and it shows a higher thermostability [14] and a broader substrate spectrum than PhyA [15,16,17].

Numerous phytase (*phy*) genes have been isolated from different organisms, some of which have been expressed in yeasts for possible industrial application [12,18,19,20,21]. *A*. *niger* phytase expressed in *Pichia* *pastoris* showed similar pH and temperature optima, molecular size, glycosylation extent and substrate specificity to the native phytase [20]. Although the phytase expression in yeast has many advantages in large-scale production, there is a disadvantage, such as that the yeast mass when taken as a diet supplement may cause indigestion or allergic reactions. Moreover, yeast cell fermentation is a very expensive process, and it needs a high level of sterility control of the production unit. The phytase expression in plants is an alternative system, which can reduce the production and formulation costs in the animal feeding industry. Besides, its expression in plant roots increased phytase activity in the rhizosphere and promoted P assimilation from soil [22,23]. For the purpose of phytoremediation, such transgenic plants expressing phytase can be applied to soils contaminated with high P-content fertilizer. To date, numerous *Aspergillus* *phy* genes have been expressed in transgenic plants [22,23,24,25,26,27,28,29,30,31,32,33]. Nevertheless, there is a demand for industrial purposes to express a phytase in a plant that has the ability to efficiently hydrolyze the substrate.

Studies about *A*. *nidulans* phytase are very limited [15,34,35] when compared with those of well-known phytases. Besides, *A*. *nidulans* phytase has never been expressed in *Escherichia coli* and plants. Prokaryotic systems lack post-translational modifications. By contrast, different heterologous protein expression systems, such as fungi and plants, are known for generating glycosylated protein. Glycosylation is an important feature of the phytase biosynthesis in fungi [21,35], providing enzyme stability and protection from proteolytic inactivation [4]*.* A serious limitation of the yeast expression system is the improper glycosylation of the proteins, as it often hyperglycosylates the recombinant proteins by too much sugar residues [36].

The first objective of the present study was to investigate whether an antibody against a recombinant protein (rPhy-E) of *A. nidulans* phytase produced in *E.* *coli* would be immunogenic with a recombinant protein (rPhy-P) expressed in a plant. In this regard, we cloned a *phy* gene from the *A. nidulans* cDNA library for expression in *E.* *coli*. The purified rPhy-E was injected into experimental animals to produce polyclonal antibody. The second objective of the present study was to evaluate the *phy* expression level and its enzyme activity in *Nicotiana benthamiana*. To do this, the *phy* gene was cloned into the plant expression vector. The resultant recombinant vector was transformed into *Agrobacterium* *tumefaciens*, and the transformed *Agrobacterium* containing the construct was infiltrated into the plant leaves by syringe. To characterize the rPhy-P, we determined its phytase activity, optimum pH, optimum temperature, glycosylation effect on the phytase activity and substrate specificities. The third objective was to assess the effects of the rPhy-P on the P excretion of experimental rats administered orally for seven days. As part of a safety evaluation for the rPhy-P, we also included toxicity and behavior studies. 

## 2. Results and Discussion

### 2.1. Overexpression of A. nidulans phy Gene in E. coli, Purification of the Phy-6x His-Tagged Fusion Protein and Production of the Recombinant Protein (rPhy-E)-Specific Antibody

**Figure 1:**
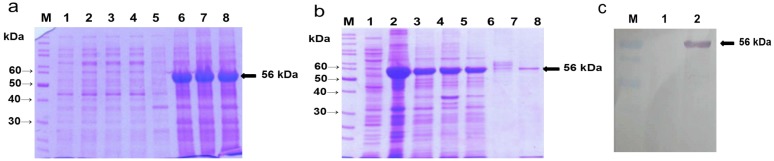
Overexpression of the *A*. *nidulans* *phy* gene in *E*. *coli*, purification of the Phy-6× His-tagged fusion protein and production of the recombinant protein (rPhy-E)-specific antibody. (**a**) After 4 h induction with IPTG, soluble (Lanes 1–4) and insoluble (Lanes 5–8) fractions of bacterial lysates were compared on 10% SDS-PAGE. A prominent band of the electrophoretic mobility (56 kDa) on SDS-PAGE was observed in the insoluble fractions of the bacterial culture. Lane M, protein marker; Lane 1, soluble protein of *E. coli* BL21(DE3) containing the pET-28a vector only; Lanes 2–4, soluble protein of *E. coli* BL21(DE3) containing pET-phy induced with IPTG at 1, 3 and 5 mM; Lane 5, insoluble protein of *E. coli* BL21(DE3) containing the pET-28a vector only; Lanes 6–8, insoluble protein of *E. coli* BL21(DE3) containing pET-phy induced with IPTG at 1, 3 and 5 mM; (**b**) The rPhy-E fused with 6× His-tag was purified from *E. coli* BL21(DE3) by affinity chromatography; (**c**) Western blot analysis of rat antibody is able to recognize the rPhy-E in total protein extracted from bacterial lysates.

The selected clones containing recombinant vector (pET-phy) were used for the protein expression analysis. After 4 h of induction at 37 °C with isopropyl-β-d-1-thiogalactopyrannoside (IPTG), soluble (Figure 1a, Lanes 1–4) and insoluble fractions (Figure 1a, Lanes 5–8) of the bacterial lysates were compared on SDS-PAGE. The highest expression of rPhy-E was achieved in the insoluble fraction, but it was not significantly influenced by IPTG concentration (Figure 1a, Lanes 6–8). A prominent band (56 kDa) on SDS-PAGE was observed in the insoluble fractions, suggesting that the cytoplasmic targeting is not preferred for rPhy-E in *E. coli* BL21 (DE3). The rPhy-E fused with 6× His-tag was purified from the bacterial lysates by affinity chromatography (Figure 1b), as described in the Experimental Section. SDS-PAGE analysis showed that the 6× His-fused rPhy-E was almost pure after final elution. Initially, the presence of the fusion protein was confirmed by western blotting using Ni-NTA AP-conjugated antibody against 6× His-tag. To produce polyclonal antibody against the rPhy-E, 56-kDa band on SDS-PAGE gel was eluted, which was used as an immunogen. Western blot analysis indicated that the rat antisera are able to recognize rPhy-E in total protein extracted from bacterial lysates (Figure 1c). In a previous study, *E. coli* was unable to express the active enzyme of the *A*. *niger* phyA gene, because of producing a nonglycosylated and intracellular inclusion protein [37].

The *A. nidulans phy* gene consists of an open reading frame of 1398 bp and encodes 466 amino acids with a deduced molecular mass of 51 kDa. We determined the nucleotide sequences of *A. nidulans phy* in pET-phy and confirmed it to have identical nucleotide and amino acid sequences of *A. nidulans* 3-PhyB (GenBank Accession Number No. XP659289). It also shares 99% identity with *A. nidulans* phytase (GenBank Accession Number No. U59803.1). However, the deduced amino acid sequences of 3-PhyB were not aligned at all with those of *A*. *niger* PhyB (GenBank Accession Number AAA02934). In addition to that, the 3-PhyB protein shared a higher percentage of amino acid identity to 3-PhyA of *A*. *oryzae* RIB40 (GenBank Accession Number XP001821210, 67%), phytase of *A*. *flavus* NRRL3357 (GenBank Accession Number XP002376973, 67%), phytase of *A*. *fumigatus* Af293 (GenBank Accession Number XP751964, 67%), 3-PhyA of *A*. *niger* CBS 513.88 (GenBank Accession Number XP001401713, 63%) and phytase of *A*. *clavatus* NRRL 1 (GenBank Accession Number XP001271757, 63%). In earlier studies, *A. nidulans* phytase was estimated as a range of molecular weight (*M*_W_) 49.0–51.8 kDa and pI values of 5.14–5.39 [14,34]. In this study, we predicted *M*_W_ and pI values of native phytase from its amino acid sequence using a web-based program. It has a theoretical *M*_W_ of 51.8 kDa and a pI value of 5.35.

**Figure 2:**
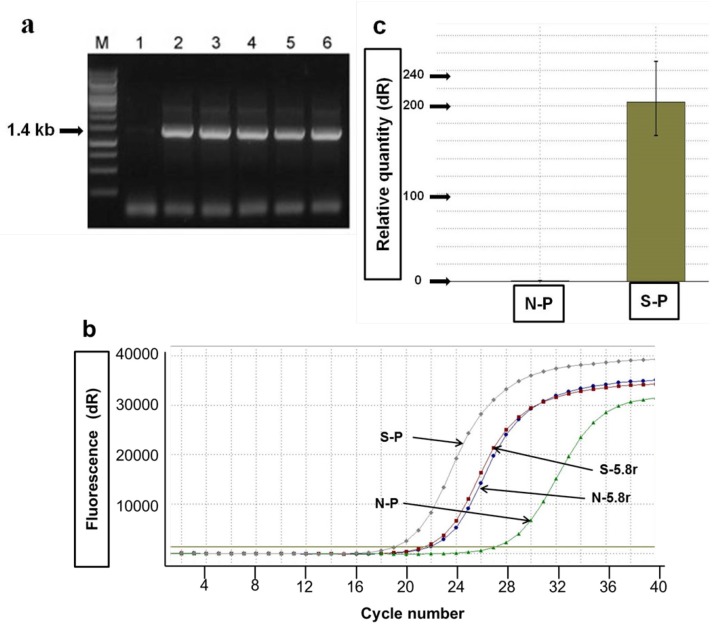
RT-PCR and quantitative real-time RT-PCR analyses. (**a**) RT-PCR analysis of *phy* mRNA expression in agro-infiltrated leaves of *N.* *benthamiana* with *Agrobacterium* containing pPZP212-phy. Lane M, 1-Kb DNA ladder; Lane 1, mock infiltrated control; Lanes 2–6, agro-infiltrated leaves at 1, 2, 3, 4 and 5 days post-infiltration (dpi); (**b**) To validate the performance of real-time RT-PCR assay, endogenous 5.8s rRNA of infiltrated leaves with *Agrobacterium* containing *phy* gene (S-5.8r) was compared with that of mock-infiltrated leaves (N-5.8r); (**c**) The relative expression level of *phy* mRNA in agro-infiltrated leaves at 5 dpi (S-P) was compared with that of *phy* mRNA in mock-infiltrated leaves (N-P).

### 2.2. Agro-Infiltration and phy Gene Expression in N. benthamiana

Agro-infiltration was performed for the transient expression of *A. nidulans* *phy* gene in *N. benthamiana*. In Figure 2a, the presence of *phy* gene mRNA in *N. benthamiana* plants was confirmed by reverse transcription polymerase chain reaction (RT-PCR). The 1.4-kb DNA fragments were amplified using total RNA extracted from agro-infiltrated leaves at 1–5 days post-infiltration (dpi), whereas there was no RT-PCR product in mock-infiltrated leaf. To measure quantitatively the *phy* gene expression in the agro-infiltrated leaves, we determined its mRNA level by quantitative real-time RT-PCR. In order to validate the real-time RT-PCR analysis with SYBR Green dye, the 5.8s rRNA of infiltrated leaves with *Agrobacterium* containing the *phy* gene was compared with the 5.8s rRNA in the mock-infiltrated leaves. As shown in Figure 2b, the result clearly exhibits the normalized reporter-dye fluorescence (dR) as a function of the cycle and indicates that the two endogenous 5.8s genes were not differently expressed in any of infiltrated leaves. The mean *C*_t_ values for the sample of infiltrated leaves with *Agrobacterium* containing *phy*gene, the reference sample for 5.8s rRNA of infiltrated leaves with *Agrobacterium* containing *phy*gene, the reference sample for 5.8s rRNA in the mock-infiltrated leaves and the negative sample of mock-infiltrated leaves were 19.11, 21.47, 21.90 and 27.21, respectively. The relative *phy* gene mRNA expression level in the agro-infiltrated leaves at 5 dpi was approximately 200-fold more than that in the mock-infiltrated leaves (Figure 2c). This suggested that the *phy* gene was highly expressed in agro-infiltrated leaf tissues, and no modification occurred at the transcriptional level.

**Figure 3:**
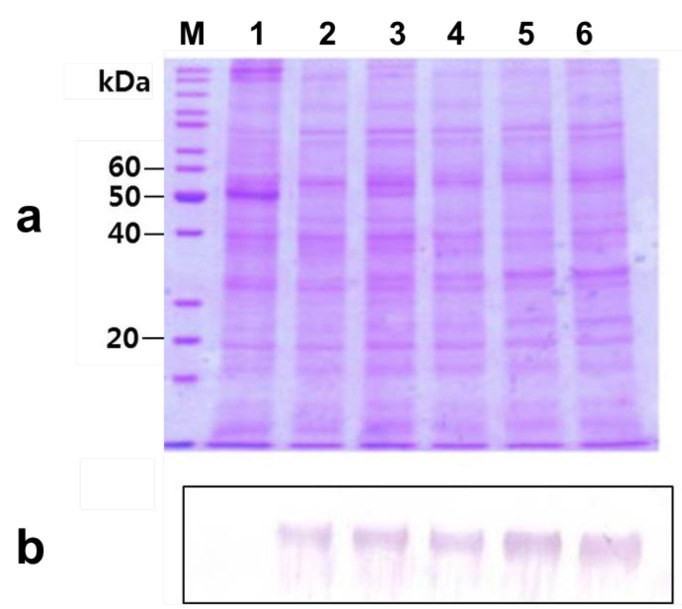
Recombinant phytase protein was identified from the extracts of agro-infiltrated leaves of *N.* *benthamiana* with *Agrobacterium* containing pPZP212-phy. (**a**) Coomassie blue-stained 10% SDS-PAGE gel; (**b**) Western blot analysis of identical gel in (**a**). Lane M, molecular weight markers; Lane 1, mock infiltrated control; Lanes 2–6, agro-infiltrated leaves at 1, 2, 3, 4 and 5 dpi.

To determine that the increased *phy* gene mRNA level leads to an increased level of protein synthesis, SDS-PAGE (Figure 3a) and western blot analysis (Figure 3b) were carried out using the total protein from agro-infiltrated plants at 1–5 dpi. As shown in Figure 3b, the plant-produced rPhy-P showed a ~62-kDa band that was recognized by a polyclonal antibody to *E*. *coli*-produced rPhy-E. It suggests that the nonglycosylated form of rPhy-E maintains an antigenic domain of native phytase. We also observed increasing accumulation of rPhy-P with increasing days after infiltration. The difference between a *M*_W_ predicted by amino acid sequences and a *M*_W_ determined by SDS-PAGE/western blot analyses reflects on the glycosylation extent of the rPhy-P. In a previous study, *A*. *nidulans* phytase expressed in *A*. *niger* was estimated to have a range of *M*_W_ 64.1~68.7 kDa by SDS-PAGE, *M*_W_ 67.4 kDa by analytical ultracentrifugation or a range of *M*_W_ 77.8~88.3 kDa by gel filtration analysis due to different glycosylation extents [35]. It suggested that the plant expression system provides less variable glycosylation extent than the fungal and yeast expression system.

The hyperglycosylation significantly contributes to the high molecular mass and heterogeneity of the recombinant protein [38]; such a protein was immunogenic in animals and humans [39,40,41,42] or reduced immunogenicity [36]. This suggests that post-translational modification can give a totally different pattern of glycosylation, resulting in undesired immunogenicity. Furthermore, the yeast hyperglycosylates N-linked sites leading to a reduction in both functional activity and receptor-binding [43,44]. The high-mannose type in yeast *N-*glycosylation mediates binding to the mannose receptors in humans and results in poor pharmacokinetic behavior [45]. On the other hand, the targeted manipulation of the plant *N*-glycosylation pathway allows the production of proteins carrying largely homogeneous, human-type oligosaccharides. Due to their rather small repertoire of glycosylation reactions, plants carry out complex *N*-glycosylation at a striking homogeneity, which makes them especially manageable to glycoengineering [46].

### 2.3. pH Dependence on the Phytase Activity of rPhy-P

**Figure 4:**
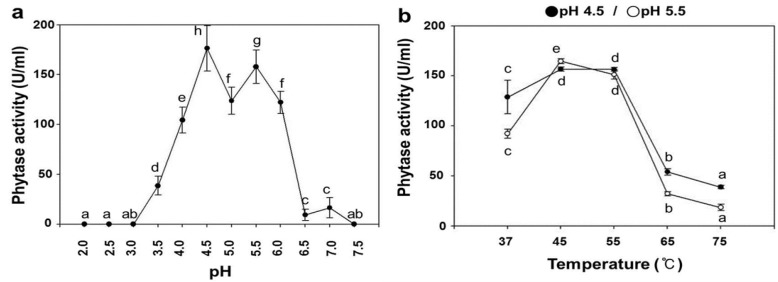
Phytase activity of *A.* *nidulans phy* gene expressed in *N.* *benthamiana* leaves by agro-infiltration at 5 dpi. (**a**) The effect of pH on phytase activity of the total protein extracts was assayed under various pH buffers at 55 °C for 30 min, and the buffers are used as follows: 50 mM glycine (pH 2.0–2.5), 50 mM sodium acetate (pH 3.5–5.5), 50 mM MES (pH 6.0–6.5) and 50 mM HEPES (pH 7.0–7.5); (**b**) The effect of temperature on the phytase activity of the total protein extracts was assayed at various temperatures, 37–75°C, for 30 min at pH 4.5 and 5.5. Data are expressed as the phytase or relative activity, and each value represents the mean ± SE (*n* = 6). Different letters indicate significant differences (*p* < 0.05).

Phytase activity is highly dependent on pH; due to this reason, we determined the optimum pH for the phytase activity of rPhy-P. To exclude endogenous phytase activity in *N*. *benthamiana* leaves, the enzyme activity values in total protein extracted from the agro-infiltrated leaves at 5 dpi were adjusted by subtraction of those values in total protein extracted from mock-infiltrated leaves over a pH range. Its highest activity was found at pH 4.5 (the first) and pH 5.5 (the second) after incubation at 55 °C for 30 min (Figure 4a) and pH 5.5 (the first) and pH 4.5 (the second) after incubation at 45 °C for 30 min (data not shown). The optimum pH range for the phytase activity of the rPhy-P was pH 4.5–5.5. This is dissimilar to previous report that showed the narrow pH profile and higher pH optimum (pH 6.5) of the *A*. *nidulans* phytase expressed in *A. niger* [15]. The differences observed in this study may be due to the expression system and the assay variation. The maximum phytase activity of the rPhy-P was 176.4 U/mL of total protein extract at pH 4.5 (55 °C) and 164.4 U/mL of total protein extract at pH 5.5 (45 °C). However, the activity was lost under the pH 3.0 or at near neutral pH.

### 2.4. Temperature Dependence on the Phytase Activity of rPhy-P

To determine the optimum temperature for the phytase activity of rPhy-P, the activity values of total protein extracted from the agro-infiltrated leaves at 5 dpi were adjusted by subtraction of those values of total protein extracted from mock-infiltrated leaves over a temperature range. The assay was performed at pH 4.5 and pH 5.5. The optimum temperatures were 45 °C at pH 5.5 and 55 °C at pH 4.5 (Figure 4b), and the latter is similar to most fungal phytases that exhibit the optimum temperature in the range of 50–60 °C [47].

**Figure 5:**
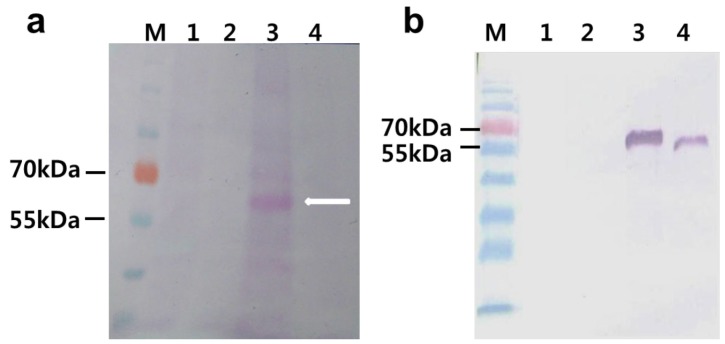
Detection of the glycosylated rPhy-P from the total protein extracts of agro-infiltrated leaf tissues with *Agrobacterium* containing pPZP212-phy at 5 dpi. (**a**) Schiff’s reagent-stained 10% SDS-PAGE gel. Lane M, molecular weight markers; Lane 1, the purified rPhy-E; Lane 2, total protein extracted from mock-infiltrated leaf; Lane 3, untreated total protein extracted from agro-infiltrated leaf tissues at 5 dpi with peptide-*N*-glycosidase F; Lane 4, treated total protein extracted from agro-infiltrated leaf tissues at 5 dpi with peptide-*N*-glycosidase F; (**b**) Western blot analysis of total protein extracted from mock-infiltrated leaf and agro-infiltrated leaf tissues at 5 dpi. Lanes 1 and 3 represent treated before and Lanes 2 and 4 represent treated after with peptide-*N*-glycosidase F.

### 2.5. Deglycosylation of the Expressed Phytase

To confirm that the rPhy-P expressed in *N*. *benthamiana* leaves is glycosylated, we performed its periodic acid-Schiff (PAS) staining and peptide-*N*-glycosidase F (PNGase-F) digestibility. The agro-infiltrated leaves at 5 dpi were chosen for the deglycosylation analysis. As shown in Figure 5a, the protein band became visible (Lane 3) in the agro-infiltrated leaf extract, but not in the *E*. *coli*-expressed rPhy-E (Lane 1), mock-infiltrated leaf extract (Lane 2) and agro-infiltrated leaf extract treated with PNGase F (Lane 4) when SDS-PAGE gel was stained with PAS reagent. The results showed that the rPhy-P is glycosylated (*M*_W_ 62 kDa, Lane 3 in Figure 5a,b). To further confirm the glycosylation of rPhy-P, total protein was digested with PNGase F, resulting in a shift of *M*_W_ from 62 to 56 kDa (Figure 5b). Thus, the deglycosylated rPhy-P was estimated to have *M*_W_ 56 kDa, indicating that the percentage of glycosylation was approximately 11%. In western blot analysis, the phytase-specific antibody recognized both glycosylated and deglycosylated forms of the rPhy-P (Lanes 3 and 4 in Figure 5b). However, the band was not detected in total protein of the mock-infiltrated leaf extract that was digested with PNGase F or not (Lanes 1 and 2 in Figure 5b). Deglycosylation studies of *Aspergilli* phytases expressed in *P. pastoris* were revealed in the reduction of the molecular size and thermostability of the secreted enzyme [18,21].

### 2.6. Glycosylation Effect on the Thermostability of the Phytase Activity of rPhy-P

**Figure 6:**
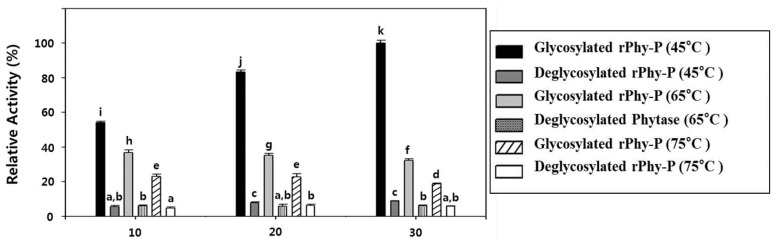
Glycosylation effect on the thermostability of the phytase activity of rPhy-P was assayed after incubation at 45, 65 and 75 °C for 10–30 min. Data are expressed as the phytase or relative activity, and each value represents the mean ± SE (*n* = 6). Different letters indicate significant differences (*p* < 0.05).

To determine the glycosylation effect on the thermostability for the phytase activity of rPhy-P, the phytase activity was measured relatively after being incubated for 10–30 min at 45, 65 and 75 °C, respectively, with and without treatment of PNGase-F (Figure 6). The rPhy-P before deglycosylation showed a relative activity of 54% after 10 min incubation at 45 °C. At this temperature, the enzyme activity increased in a time-dependent manner and reached 100% after 30 min of incubation. Although the enzyme activity significantly dropped to 37% and 23% after 10 min of incubation at 65 and 75 °C, respectively, it still remained more than 32% and 18% of activity after 30 min of incubation at each temperature. However, the deglycosylated rPhy-P showed the rapid reduction of the relative enzyme activity after 10 min of incubation at 45 °C (6%), 65 °C (6%) and 75 °C (5%). Indeed, the deglycosylation caused a substantial reduction of phytase thermostability. The result confirms that the glycosylation provides the thermostability for the phytase activity of rPhy-P.

### 2.7. Substrate Specificities of Phytase Expressed in Agro-Infiltrated Leaves

**Figure 7:**
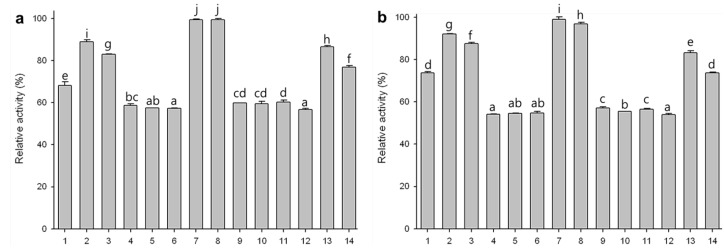
Substrate specificities of rPhy-P at 45 °C (**a**) and 55 °C (**b**). All substrates were used at a concentration of 2 mM. 1, AMP; 2, ATP; 3, ADP; 4, ethanolamine phosphate; 5, *O*-phospho-l-serine; 6, β-glycerophosphate; 7, tripolyphosphate; 8, pyrophosphate; 9, pyridoxal-5-phosphate; 10, fructose-6-phosphate; 11, glucose-6-phosphate; 12, glucose-1-phosphate; 13, acetyl phosphate; 14, sodium phytate. Data are expressed as the relative activity and each value represents the mean ± SE (*n* = 3). Different letters indicate significant differences (*p* < 0.05).

To determine the substrate specificities of the rPhy-P, we investigated the specific activities of the total protein extracts with a series of phosphate compounds. Initially, mock-infiltrated controls were measured for determining endogenous phytase activity for each substrate at 45 and 55 °C. As shown in Figure 7, the rPhy-P displayed broad substrate specificities at both temperatures, especially higher enzyme activities for AMP, ATP, ADP, tripolyphosphate, pyrophosphate, acetyl phosphate and sodium phytate. Among those substrates, the highest activity was observed with tripolyphosphate.

**Figure 8:**
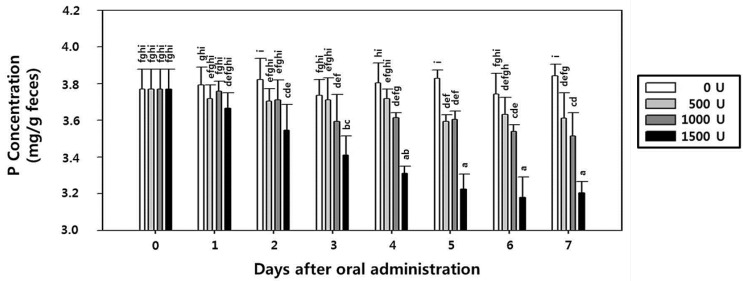
Fecal phosphorus (P) concentration in rats orally administered rPhy-P for seven days. Extractable P was extracted from 1 g feces using Mehlich III solution and determined by ascorbic acid colorimetry. Each value represents the mean ± SE (*n* = 3). Different letters indicate significant differences (*p* < 0.05).

### 2.8. Effect of rPhy-P on Phosphorus Reduction and Toxicity in Rat Fecal Samples

To evaluate the P content reduction, rats were orally administrated with rPhy-P once a day. Rats administered with three different doses (500, 1000 and 1500 U/kg body weight (bw)/day) showed significant reduction of fecal P excretion when compared with control group rats. Relative to the control group, extractable P content in fecal samples was reduced to 4.2%, 6.8% and 16%, respectively, for the given doses of rPhy-P (Figure 8). In addition, the rPhy-P under the present experimental condition did not result in toxicologically significant changes at any groups in bw (Supplementary Table S1), organ weights (Supplementary Table S2), mortality and clinical signs, such as salivation, prone position, decrease of locomotive activity, loss of fur, bite wounds, scratch wounds, lacrimation and closed eyes (Supplementary Table S3). This suggests that the rPhy-P can be used safely as feeding supplements to livestock, and it will reduce the environmental impact of P from manure.

## 3. Experimental Section

### 3.1. Computer-Aided Analyses

The amino acid sequence of the *A. nidulans phy* gene was used to determine the identity of the protein using the BLASTp program [48] and to predict theoretical *M*_W_ and pI using the Compute pI/*M*_W_ tool [49].

**Table 1:** Primers used in this study.

Template	Primer	Nucleotide Sequences (5'→3')
cDNA libraries	phy1-F ^1^	GAATTCGCTTTTTTCACGGTCGCTCTT
	phy1-R ^2^	GTCGACTAGGGTAAAACAAGTCTTCCA
pRTL2	M1-RTL-F ^3^	GAAAATTTTCACCATTTACGAACGATAGCCGCGGCACTCATC
	M1-RTL-R ^4^	GATGAGTGCCGCGGCTATCGTTCGTAAATGGTGAAAATTTTC
pET-phy	phy2-F ^5^	CCGCGGCAATGGCTTTTTTCACG
	phy2-R ^6^	GGTACCTTATAGGGTAAAACAAGTCTTCCA
pRTL2-MS-Phytase	M2-RTL-F ^7^	TTACGAACGATAGCCATGGCAATGGCTTTTTTC
	M2-RTL-R ^8^	GAAAAAAGCCATTGCCATGGCTATCGTTCGTAA
pPZP212-phy	phy3-F	CCGCGGCATCTCATGTTTGGGGT
	phy3-R	TCTAGAGGGGCTTCCGGCACC
*N*. *benthamiana* total RNA (mock and agroinfitrated)	phy-domain-F ^9^	CAGAGTCGAAGAGTAAGGCGTAC
	phy-domain-R ^9^	GTAGAACTTGGCACCCGAATCAAC
*N*. *benthamiana* total RNA (mock and agroinfitrated)	5.8s rRNA-F ^10^	GTGATCTGTGGAAGGATCATTGTCG
	5.8s rRNA-R ^10^	CGTTAATCATCCGACACGAACGC

^1^ Forward primer contains *EcoR*I site (underline); ^2^ reverse primer contains *Sal*I site (underline); ^3,4^ primers for the site-directed mutagenesis, resulting in the change from *Nco*I site to *Sac*II site (underline represents the modified sequence); ^5^ forward primer contains *Sac*II site (underline); ^6^ reverse primer contains *Kpn*I site (underline); ^7,8^ primers for the site-directed mutagenesis, resulting in the recovery of start codon (underline represents the modified sequence); ^9^ phytase domain detection primers for real-time PCR; ^10^ 5.8s rRNA detection primers for real-time PCR.

### 3.2. PCR and Cloning of the phy Gene of A. nidulans into pET28a

Using PCR, a full-length ORF encoding the *phy* gene was synthesized from *A. nidulans* cDNA library [50] that was kindly gifted by Prof. Suhn-Kee Chae (Pai Chai University, Deajeon, Korea). Primers (phy1-F and phy1-R) in Table 1 were designed according to the nucleotide sequences of *A. nidulans* 3-PhyB in the NCBI database. PCR was performed in 50 µL of the reaction mixture consisting of 5 µL of 10× PCR buffer (100 mM Tris-HCl, pH 8.3, 500 mM KCl), *A. nidulans* cDNA library (10 ng), 4 µL of 2.5 mM dNTP mixture, 1 µL of primer each (50 pM), 3–9 µL of 25 mM MgCl_2_ and 0.5 µL (1 U) of Ex-Taq polymerase (Takara Bio, Otsu, Shiga, Japan). PCR was performed using a thermal cycler (Gene Cycler, Bio-Rad Lab., Hercules, CA, USA) with an initial denaturation at 95 °C for 2 min and followed by 35 cycles of denaturation at 94 °C for 1 min, annealing at 58 °C for 2 min, extension at 72 °C for 3 min and an additional 1 cycle of final extension at 72 °C for 7 min. Purification of PCR products was done using PCR quick-spin™ (iNtRON Biotech., Seongnam, Korea) according to the manufacturer’s protocol. The amplified 1398-bp fragments were digested with *EcoR*I and *Sal*I and then ligated into the pET28a (Merck Millipore, Darmstadt, Germany) vector, which was digested with the same enzymes. The ligation mixture was incubated at 37 °C for 4 h and then used to transform in *E.* *coli* BL21 cells by heat shock at 42 °C for 1 min. Colonies were selected by the restriction enzyme digestion and PCR analysis. The pET28a vector containing the target insert (pET-phy) was purified from the selected colonies and then sent to a commercial sequencing lab (COSMO genetech, Seoul, Korea) to determine its nucleotide sequence. The selected clones were used for protein expression studies.

### 3.3. Expression and Purification of His-Tagged Recombinant Protein pET28a

The recombinant vector (pET-phy) was transformed into *E. coli* BL21 (DE3). Expression of 6× His-fused recombinant protein was carried out according to the manufacturer’s protocol (Qiagen, Hilden, Germany). A single colony was grown in Luria-Bertani (LB) medium containing kanamycin (30 μg/mL) to an OD_600_ of 0.6. Different concentrations of IPTG (final concentration of 1, 3 and 5 mM) were treated, respectively, into the medium. Bacterial cells were further incubated at 37 °C for 4 h with shaking. After centrifugation at 10,000 rpm for 5 min, the resulting pellets were resuspended in lysis buffer (10 mM imidazole, 300 mM NaCl, 50 mM NaH_2_PO_4_, pH 8.0) and sonicated 4 times (30 s/each). After centrifugation, total protein collected from the lysate was designated as a soluble fraction. The resultant pellets were resuspended in phosphate-buffered saline buffer (PBS; 140 mM NaCl, 10 mM Na_2_HPO_4_, 2.7 mM KCl, and 1.8 mM KH_2_PO_4_, pH 7.4), which was designated as an insoluble fraction. Total protein (20 μg) was analyzed by SDS-PAGE according to Laemmli [51]. Western blot analysis was performed to detect 6× His-tagged recombinant protein using Ni-nitrilotriacetic acid (NTA) AP-conjugated antibody against 6× His-tag (Qiagen, Hilden, Germany) at a dilution of 1:5000 (*v*/*v*). The target protein was purified from *E*. *coli* cells (100 mL culture) using Ni-NTA Superflow under denaturing conditions (8 M urea) as described in the manufacturer’s manual. For the elution of 6× His-fused recombinant protein under denaturing conditions, we used the elution buffer containing 100 mM NaH_2_PO_4_, 10 mM Tris-HCl and 8 M urea. The relative efficiency of protein extraction was examined by SDS-PAGE of supernatants (“cleared lysates”) after resuspension of an aliquot in Laemmli’s loading buffer.

### 3.4. Production of rPhy-E Specific Polyclonal Antibody

The column-purified 6× His-fused protein was further purified by SDS-PAGE through a 10% preparative gel (Bio-Rad Lab., Hercules, CA, USA). Prior to injection, the purified rPhy-E was dialyzed twice against 1.5 L PBS (each for 1.5 h at 4 °C) and then used as an immunogen for the preparation of polyclonal rat antisera. The immunization was performed in the Experimental Animal Handling Facility in the Department of Biology and Medicinal Science at Pai Chai University, Daejeon, Korea, in compliance with ethical regulations. Injections were given to three rats once every 2 weeks at a concentration of 100 μg in 500 μL PBS. The rats received rPhy-E with Freund’s complete adjuvant for the primary injection and Freund’s incomplete adjuvant for the booster injections. A total of three injections were given. The rats were sacrificed, and blood was collected from the rats within 7 days after the booster injection. The polyclonal antisera against rPhy-E were used to perform western blot analysis. Total protein collected from the bacterial lysates was separated by SDS-PAGE gel and transferred to a nitrocellulose membrane that was blocked with 5% (*w*/*v*) nonfat dry milk in PBS. The membrane was probed with polyclonal antisera against rPhy-E as a primary antibody at a dilution of 1:1000 (*v*/*v*) in PBS for 1 h at room temperature and successively with goat anti-rat IgG-alkaline phosphatase conjugate (Sigma-Aldrich Co., St. Louis, MO, USA) as a secondary antibody at a dilution of 1:7000 (*v*/*v*) in PBS for 1 h at room temperature. The membrane was developed by a conventional method using nitroblue tetrazolium (NBT) and 5-bromo-4-chloro-3-indolylphosphate (BCIP) (Sigma-Aldrich Co., St. Louis, MO, USA) in 100 mM Tris, 100 mM NaCl and 5 mM MgCl_2_ at pH 9.5.

### 3.5. PCR Amplification of phy Gene and Vector Construction for Plant Expression

To introduce the *A. nidulans* *phy* gene into a shuttle vector (pRTL2), we designed site-directed mutagenesis (SDM)-PCR twice in the cloning strategy (Supplementary Figure S1a,b). SDM-PCR was performed using PfuUltra™ DNA polymerase (Agilent Tech., Santa Clara, CA, USA) for mutagenic primer-directed replication of both plasmid strands with the highest fidelity. The *Nco*I site in the leader sequences of pRTL2 contains ATG nucleotides that were used for the translation start codon. We modified the vector sequences of pRTL2, because the *phy* gene also contains three *Nco*I sites in the coding region (Supplementary Figure S1c). The *Nco*I site was changed to the *Sac*II site (5'-CCATGG-3' → 5'-CCGCGG-3'; the underline represents the modified sequence) by the primary SDM-PCR. To modify the pRTL2 vector, we synthesized two complementary oligonucleotides that contained the desired mutation flanked by unmodified nucleotide sequences (Table 1). The primary SDM-PCR was performed in 50 µL of reaction mixture consisting of 10× PCR buffer, pRTL2 DNA (10 ng), 4 µL of 2.5 mM dNTP mixture, 1 µL of 50 pM primers of forward (M1-RTL-F) and reverse (M1-RTL-R) primer each and 1 µL (2.5 U) of PfuUltra™ DNA polymerase. PCR was performed in a thermal cycler with initial denaturation at 95 °C for 30 s, followed by 15 cycles of denaturation at 95 °C for 30 s, annealing at 55 °C for 1 min and extension at 68 °C for 5 min. After PCR, the amplified product was treated with *Dpn*I, which is specific for the digestion of the *in vivo*-methylated parental template and hybrid DNA. The amplified product was treated with *Dpn*I (10 U) and *Pfu* DNA polymerase (2.5 U); then, the mixture was incubated at 37 °C for 30 min and shifted to 72 °C for an additional 30 min. Extension of the primers generated a mutated plasmid (pRTL2-MS) containing staggered nicks, which were repaired after competent cell transformation.

To insert the target gene into pRTL2-MS, a full-length *phy* was synthesized with primers (phy2-F and phy2-R) in Table 1. PCR mixture included 5 µL of 10× PCR buffer, 4 µL of 2.5 mM dNTP mixture, 1 µL of primer each (stock concentration, 50 pM), 0.5 µL (2.5 U) of *Taq* DNA polymerase (Takara, Japan), pET-phy DNA (10 ng) and sterile distilled water added to make up 50-µL final volumes. The PCR was performed in a thermal cycler with an initial denaturation at 95 °C for 2 min, followed by 35 cycles of 94 °C denaturation for 1 min, annealing temperature 59 °C for 2 min, 72 °C extension for 3 min and 1 cycle of a 7-min final extension at 72 °C. Purification of the PCR product was done using PCR quick-spin™ according to the manufacturer’s protocol. The purified PCR product was digested with *Sac*II and *Kpn*I and cloned into the pRTL2-MS that was digested with the same restriction enzymes. The resultant plasmid was subsequently transformed into *E. coli* XL1-Blue. Transformed colonies were selected on LB media containing carbenicillin (50 µg/mL) and used for the plasmid isolation. The presence of the *phy* gene in the resultant plasmid pRTL2-MS-phytase was confirmed by restriction enzyme digestion (*Sac*II and *Kpn*I) and PCR amplification using phy2-F and phy2-R primers. The resultant vector pRTL2-MS-phytase possessed an expression cassette containing *Cauliflower mosaic virus* (CaMV) 35S promoter (P35S), a TEV leader sequence, *phy* sequence and a 35S terminator (T35S).

The pRTL2-MS-phytase was used as a template to perform SDM for recover the start codon of *phy* using mutagenic primers (M2-RTL-F and M2-RTL-R) in Table 1, resulting the change of *Sac*II to *Nco*I site. The secondary SDM-PCR was performed in 50 µL of reaction mixture consisting of 10× PCR buffer (Agilent Tech., Santa Clara, CA, USA), pRTL2-MS-phytase DNA (10 ng), 1 µL of 2.5 mM dNTP, 1 µL of 50 pM of primer each and 1 µL (2.5 U) of PfuUltra™ DNA polymerase. PCR was carried out in a thermal cycler with an initial denaturation at 95 °C for 2 min, followed by 20 cycles of 95 °C denaturation for 30 s, annealing temperature 64 °C for 1 min, 72 °C extension for 2 min and an additional 1 cycle of 7 min final extension at 72 °C. The amplified product was treated with *Dpn*I as mentioned above and the resultant plasmid pRTL2-phytase transformed into *E. coli* XL1-Blue competent cells at 42 °C for 1 min. The expression cassette region excised from pRTL2-phytase using *Hind*III, which was ligated into the T-DNA region of a binary vector, pPZP212, to produce pPZP212-phy. The ligation mixture was transformed into the *E. coli* XL1-Blue competent cell by the heat-shock method. The transformants were selected on LB media containing spectinomycin (100 μg/mL), and the selected colonies were used for plasmid isolation. The presence of the *phy*-inserted cassette region was determined by *Hind*III digestion and PCR amplification using M2-RTL-F and M2-RTL-R primers.

### 3.6. Agrobacterium Transformation, Agro-Infiltration and Analysis by RT-PCR and Quantitative Real-Time RT-PCR

*Agrobacterium tumefaciens* strain C58C1 cells were transformed with pPZP212-phy by electroporation. The transformed cells were selected on LB media containing spectinomycin (100 μg/mL), streptomycin (100 μg/mL), gentamycin (50 μg/mL) and rifampicin (50 μg/mL). The presence of the gene expression cassette was determined by *Hind*III digestion and PCR amplification using phy3-F and phy3-R primers (Table 1). Overnight culture of *Agrobacterium* carrying pPZP212-phy was centrifuged at 6000× *g* for 3 min, and the resultant pellet was resuspended in a solution containing 10 mM morpholinepropanesulfonic acid (MES, pH 5.5), 10 mM MgCl_2_ and 100 μM acetosyringone. Bacterial suspension containing the construct was infiltrated into the first two leaves of young *N. benthamiana* with a 3-mL needleless syringe. The infiltrated plants were kept in a growth room (12-h day length and 26 °C). Agro-infiltrated *N*. *benthamiana* leaves were harvested at 1–5 dpi. Total RNA was extracted from the homogenized leaves using the RNeasy plant mini kit (Qiagen, Hilden, Germany) according to the manufacturer’s instruction. RT reaction was performed in the reaction volume of 20 µL containing 500 ng of total RNA, 50 mM Tris-HCl (pH 8.3), 75 mM KCl, 10 mM DTT, 3 mM MgCl_2_, 1 mM dNTP, 20 pM of phy3-R primer, 20 U of RNase inhibitor (Takara Bio, Otsu, Shiga, Japan) and 200 U Moloney murine leukemia virus (M-MLV) reverse transcriptase (Promega, Madison, WI, USA). The thermal cycler was programmed for 1 RT cycle at 45 °C for 30 min. For PCR amplification, 10 µL of the RT product were added to 40 µL of reaction mixture containing 10 mM Tris-HCl (pH 8.3), 50 mM KCl, 1.5~4.5 mM MgCl_2_, 2.5 U Taq polymerase, 2.5 mM dNTP and 50 pM of phy3-F and phy3-R primers. The thermal cycler was programmed for 35 cycles of template denaturation at 94 °C for 1 min, primer annealing at 61 °C for 2 min, and DNA synthesis at 72 °C for 3 min. For quantitative real-time RT-PCR, *phy* mRNA and 5.8s rRNA were reversed from total RNA of agro-infiltrated leaves at 5 dpi and mock-infiltrated leaves to cDNA by M-MLV reverse transcriptase using the primers shown in Table 1. The endogenous 5.8s rRNA was used as an internal control for normalization in all experimental groups. Real-time PCR was carried out in 96-well plates by the Stratagene Mx3005P cycler. The reaction mixture (20 µL) contained 1 ng cDNA, 10 µL of 2× SYBR Green QPCR Master Mix (Agilent Tech., Santa Clara, CA, USA) and 1 µL of primer pairs at 10 pM. Conditions were set as follows: initial denaturation at 95 °C for 15 min, 40 cycles of denaturation at 95 °C for 10 s and annealing and extension at 60 °C for 40 s. The experiments were analyzed with auto-baseline and manual thresholds chosen from the exponential phase of the PCR amplification. After the data analysis, the Ct number and DeltaRn were used for statistical analyses. The expression level of *phy* gene was determined by 3 replicates of one sample and 3 independent experiments. Data were analyzed using Mxpro software and the comparative threshold cycle (2^−ΔΔ*C*t^) method [52].

### 3.7. SDS-PAGE, Western Blot and Deglycosylation Analysis of Protein Extracts

The agro-infiltrated leaves were harvested at 1–5 dpi, and total protein extracts were prepared by grinding the leaves in liquid nitrogen and dissolved in PBS, pH 7.4. After centrifugation for 20 min (12,000× *g*, 4 °C), the supernatant was collected, and its protein concentration was estimated using a protein assay kit (Bio-Rad, Hercules, CA, USA). A mock-infiltrated control was performed simultaneously, for that infiltration buffer alone was used. Total protein (20 μg) was subjected to 10% SDS-PAGE. The glycan moiety of the rPhy-P was glycol-stained by PAS reagent, as previously described [53]. Western blot analysis was performed as described in the Section 3.4. Deglycosylation of rPhy-P was performed by using PNGase F (Sigma-Aldrich Co., St. Louis, MO, USA) according to the manufacturer’s instructions. Briefly, 45 µL of total protein extracts (20 μg) at 5 dpi in 50 mM sodium phosphate buffer (pH 7.5) were mixed with 5 µL of denaturation solution (0.2% SDS, 100 mM 2-mercaptoethanol). After boiling for 10 min, the mixture was treated with 15% (*v*/*v*) Triton X-100 and 2.5 U of PNGase F. After incubation at 37 °C for 3 h, the mixture was subjected to 10% SDS-PAGE and western blot.

### 3.8. Phytase Assay and Substrate Specificities

Phytase activity was assayed by the ferrous sulfate-molybdenum blue method [54]. Briefly, 75 μL of total protein (10 μg) were incubated with 300 μL of 2 mM sodium phytate and 0.1 mM CaCl_2_ at 45 and 55 °C, respectively, for 30 min. The reaction was stopped by adding 375 μL of 5% trichloroacetic acid. The released inorganic phosphate was analyzed by adding 750 μL of a coloring reagent, including 4 volumes of 1.5% (*w*/*v*) ammonium molybdate solution in 5.5% (*w*/*v*) sulfuric acid solution and 1 volume of 2.7% (*w*/*v*) ferrous sulfate solution. The mock-infiltrated control was performed identically as described above. The absorbance of the supernatant of each sample was read at 700 nm after appropriate dilution, and the value was adjusted by subtraction of absorbance of mock-infiltrated leaf extracts at 700 nm. Enzyme activity was calculated as unit (U) per mL protein extract, where 1 U liberates 1 μmol phosphate from phytate per min under the assay conditions. A standard curve was prepared for the range of 0–666.7 μM of K_2_HPO_4_. All assays were carried out in triplicate along with appropriate buffer and reagent controls. To determine the relative substrate specificities, the sodium phytate in the assay mixture was replaced with different phosphate compounds at a concentration of 2 mM and assayed at 45 and 55 °C, respectively, under pH 4.5.

### 3.9. Determination of Optimal pH and Temperature for Phytase Activity

Agro-infiltrated leaves (0.1 g) at 5 dpi were homogenized in 1 mL of various buffers over a pH range from 2.0 to 7.5, respectively. Buffer systems were used to cover the pH range, such as 50 mM glycine-HCl for pH 2.0–2.5, 50 mM sodium acetate for pH 3.5–5.5, 50 mM MES for pH 6.0–6.5 and 50 mM HEPES for pH 7.0–7.5. Phytase activity was measured using 2 mM sodium phytate as a substrate at 45 and 55 °C, respectively, for 30 min. In addition, the enzyme activity was also measured at different temperature (37–75 °C) for 30 min under pH 4.5 and pH 5.5, respectively, to obtain the optimum temperature.

### 3.10. Determination of Thermostability before and after Deglycosylation

In order to determine thermostability, the total protein was incubated at 45, 55, 65 and 75 °C for 10, 20 and 30 min, respectively, with and without treatment of PNGase-F. After cooling at room temperature, the phytase activity in total protein was measured as described above. Deglycosylation of the total protein was done as described in Section 3.7, except the denaturation condition.

### 3.11. Experimental Animal Trial and Design

All experiments, including the treatment and maintenance of experimental animals, were approved by the Ethical Committee for Animal Care in the Department of Biology and Medicinal Science of Pai Chai University. Male Sprague-Dawley rats (5 weeks old) of 136–173 g bw were individually housed in polycarbonate cages in a temperature (22 °C) and humidity (55%) controlled room with a 12-h light:12-h dark cycle. Rats were fed standard rat diets (Jeil Feed Company, Ltd., Daejeon, Korea) containing wheat bran, soybean meal-dehulled, wheat, corn, fish meal, limestone, vitamin-mineral premix, dl-Methionine, l-lysine-HCl, salt, lard/tallow, potassium phosphate and tribasic (the composition: 22.5% crude proteins, 3.5% crude lipids, 0.7% calcium (Ca), 1% total P, 7% crude fiber, 10% crude ash). Diets were supplied as a pellet (2.2 g dry weight/kg bw) 5-times daily during the entire experimental period of 7 days. Rats had free access to demineralized drinking water. The experimental rats were divided equally into 4 groups (A, B, C and D) to evaluate the excreted P content in fecal samples. Rats from group A were administered orally with sterilized PBS only. Rats in the B, C and D groups were orally administered once daily through an intragastric tube with rPhy-P in PBS at dose levels of 500, 1000 and 1500 U/kg bw, respectively. During the experimental period, bw and organ weights, mortality and clinical signs were recorded daily. External eye examinations were performed during the pretest period, and all animals in each group were briefly examined via both external and fundus examinations using an ophthalmoscope (Heine Mini3000, Herrsching, Germany). All experimental rats were sacrificed at the end of the treatment period. Target organs (heart, liver, spleen, brain, lung and left and right kidneys) were collected, and each weight was measured.

### 3.12. Determination of Phosphorus (P) in Fecal Samples

Extractable P in fecal samples were determined based on the observation that ammonium molybdate and potassium antimony tartrate react with dilute ortho-P solutions in an acid medium to form an antimony-phosphomolybdate complex, according to Murphy and Riley [55]. According to the modified Mehlich III method [56], fecal samples were extracted by shaking of 1 g dry feces with 20 mL Mehlich III extracting solution (200 mM acetic acid, 250 mM ammonium nitrate, 15 mM ammonium fluoride, 13 mM nitric acid, 1 mM EDTA) for 10 min at room temperature and filtering through Whatman No. 2 filter paper. Its P concentration was analyzed by ascorbic acid colorimetry using a blank and standards prepared in the Mehlich III extracting solution. To prepare samples, 4.0 mL reagent B (1.584 g l-ascorbic acid and 300 mL of reagent A; 6 g ammonium molybdate, 0.146 g antimony potassium tartrate, 72 mL sulfuric/1 L deionized water) and 19 mL deionized water were added to 2 mL of each extract. Standards consisting of 5 mL of each standard P solution (0.1 ppm to 100 ppm P), 4 mL Reagent B and 16 mL deionized water and a 0.0 ppm P standard consisting of 4.0 mL Reagent B and 21 mL deionized water were also prepared. Samples and standard solution were allowed 30 min for color development, and the absorbance at 880 nm was measured with a Biochrom Libra S22 UV/VIS spectrophotometer (Cambridge, UK).

### 3.13. Statistical Analysis

Data were analyzed by a one-way analysis of variance (ANOVA) for statistical significance by SAS (Statistical Analysis System, version 14.0, Cary, NC, USA) and comparisons between the mean values of treatments using Duncan’s multiple range tests with significance at *p* < 0.05. Standard errors of the means were also calculated and are marked in Figure and Table.

## 4. Conclusions

The *A. nidulans* *phy* gene was highly expressed in *E. coli*. Using the purified rPhy-E, we produced polyclonal antibody that was used for the detection of recombinant protein, rPhy-P, carrying phytase activity in the plant. A thermostable phytase of *A. nidulans* was transiently expressed with *M*_W_ 62 kDa in *N. benthamiana* by agroinfiltration. The rPhy-P showed glycosylation, optimum pH range (pH 4.5–5.5), optimum temperature range at 45~55 °C, thermostability and broad substrate specificities. Besides, the rPhy-P was effective at reducing phosphorus excretion in rat feces when administered orally for seven days. However, the deglycosylation of rPhy-P caused a significant reduction in enzyme thermostability. This is the first report of the expression of an *A. nidulans* recombinant phytase in a plant system.

## Supplementary Materials

Supplementary materials can be found at http://www.mdpi.com/1422-0067/15/9/15571/s1.

## Supplementary Files

Supplementary File 1

## References

[1] Lei X.G., Porres J.M., Mullaney E.J., Brinch-Pedersen H., MacCabe A.P., Polaina J. (2007). Phytase: Source, structure and application. Industrial Enzymes Structure, Function and Applications.

[2] Singh B., Gotthard Kunze G., Satyanarayana T. (2011). Developments in biochemical aspects and biotechnological applications of microbial phytases. Biotechnol. Mol. Biol. Rev..

[3] Al-Asheh S., Duvnjak Z. (1994). Characteristics of phytase produced by *Aspergillus carbonarius* NRC 401121 in canola meal. Acta Biotechnol..

[4] Casey A., Walsh G. (2003). Purification and characterization of extracellular phytase from *Aspergillus niger* ATCC 9142. Bioresour. Technol..

[5] Fujita J., Yamane Y.-I., Fukuda H., Kizaki Y., Wakabayashi S., Shigeta S., Suzuki O., Ono K. (2003). Production and properties of phytase and acid phosphotase from a sake koji mold, *Aspergillus oryzae*. J. Biosci. Bioeng..

[6] Pasamontes L., Haiker M., Wyss M., Tessier M., van Loon A.P. (1997). Gene cloning, purification, and characterization of a heat-stable phytase from the fungus *Aspergillus fumigatus*. Appl. Environ. Microbiol..

[7] Ullah A.H.J., Cummins B.J. (1987). Purification, *N*-terminal amino acid sequence and characterization of the pH 2.5 optimum acid phosphatase (E.C. 3.1.3.2) from *Aspergillus ficuum*. Prep. Biochem..

[8] Ullah A.H.J., Cummins B.J. (1988). *Aspergillus ficuum* extracellular pH 6.0 optimum acid phosphatase: Purification, *N*-terminal amino acid sequence, and biochemical characterization. Prep. Biochem..

[9] Ullah A.H.J., Gibson D.M. (1987). Extracellular phytase (E.C. 3.1.3.8) from *Aspergillus ficuum* NRRL 3135: purification and characterization. Prep. Biochem..

[10] Vats P., Banerjee U.C. (2005). Biochemical characterisation of extracellular phytase (myo-inositol hexakisphosphate phosphohydrolase) from a hyper-producing strain of *Aspergillus niger* van Teighem. J. Ind. Microbiol. Biotechnol..

[11] Zhang G.Q., Dong X.F., Wang Z.H., Zhang Q., Wang H.X., Tong J.M. (2010). Purification, characterization, and cloning of a novel phytase with low pH optimum and strong proteolysis resistance from *Aspergillus ficuum* NTG-23. Bioresour. Technol..

[12] Lei X.G., Porres J.M. (2003). Phytase enzymology, applications, and biotechnology. Biotechnol. Lett..

[13] Wodzinski R.J., Ullah A.H.J. (1996). Phytase. Adv. Appl. Microbiol..

[14] Wyss M., Pasamontes L., Remy R., Kohler J., Kusznir E., Gadient M., Muller F., van Loon A.P.G.M. (1998). Comparison of the thermostability properties of three acid phosphatases from molds: *Aspergillus fumigatus* phytase, *A. niger* phytase, and *A. niger* pH 2.5 acid phosphatase. Appl. Environ. Microbiol..

[15] Wyss M., Brugger R., Kronenberger A., Remy R., Fimbel R., Oesterhelt G., Lehmann M., van Loon A.P.G.M. (1999). Biochemical characterization of fungal phytases (myo-inositol hexakisphosphate phosphohydrolases): Catalytic properties. Appl. Environ. Microbiol..

[16] Kostrewa D., Wyss M., D’Arcy A., van Loon A.P.G.M. (1999). Crystal structure of *Aspergillus niger* pH 2.5 acid phosphatase at 2.4 Å resolution. J. Mol. Biol..

[17] Mullaney E.J., Ullah A.H.J. (2003). The term phytase comprises several different classes of enzymes. Biochem. Biophys. Res. Commun..

[18] Han Y., Lei X.G. (1999). Role of glycosylation in the functional expression of an *Aspergillus niger* phytase gene (*phyA*) in *Pichia pastoris*. Arch. Biochem. Biophys..

[19] Han Y., Wilson D.B., Lei X. (1999). Expression of an *Aspergillus niger* phytase gene (*phyA*) in *Saccharomyces cerevisiae*. Appl. Environ. Microbiol..

[20] Rodriguez E., Mullaney E.J., Lei X.G. (2000). Expression of *Aspergillus fumigatus* phytase gene in *Pichia pastoris* and characterization of the recombinant enzyme. Biochem. Biophys. Res. Commun..

[21] Guo M., Hang H., Zhu T., Zhuang Y., Chu J., Zhang S. (2008). Effect of glycosylation on biochemical characterization of recombinant phytase expressed in *Pichia pastoris*. Enzym. Microb. Technol..

[22] Liu J.F., Wang X.F., Li Q.L., Li X., Zhang G.Y., Li M.G., Ma Z.Y. (2011). Biolistic transformation of cotton (*Gossypium hirsutum* L.) with the *phyA* gene from *Aspergillus ficuum*. Plant Cell Tissue Organ Cult..

[23] Richardson A.E., Hadobas P.A., Hayes J.E. (2001). Extracellular secretion of *Aspergillus* phytase from *Arabidopsis* roots enables plants to obtain phosphorus from phytate. Plant J..

[24] Brinch-Pedersen H., Hatzack F., Sørensen L.D., Holm P.B. (2003). Concerted action of endogenous and heterologous phytase on phytic acid degradation in seed of transgenic wheat (*Triticum aestivum* L.). Transgenic Res..

[25] Chen R., Xue G., Chen P., Yao B., Yang W., Ma Q., Fan Y., Zhao Z., Tarczynski M.C., Shi J. (2008). Transgenic maize plants expressing a fungal phytase gene. Transgenic Res..

[26] Drakakaki G., Marcel S., Glahn R.P., Lund E.K., Pariagh S., Fisher R., Christou P., Stoger E. (2005). Endosperm-specific co-expression of recombinant soybean ferritin and *Aspergillus* phytase in maize results in significant increases in the levels of bioavailable iron. Plant Mol. Biol..

[27] George T.S., Richardson A.E., Hadobas P.A., Simpson R.J. (2004). Characterisation of transgenic *Trifolium subterraneum* L. which expresses *phyA* and releases extracellular phytase: growth and P nutrition in laboratory media and soil. Plant Cell Environ..

[28] George T.S., Simpson R.J., Hadobas P.A., Richardson A.E. (2005). Expression of a fungal phytase gene in *Nicotiana tabacum* improves phosphorus nutrition of plants grown in amended soils. Plant Biotechnol. J..

[29] Lucca P., Hurrell R., Potrykus I. (2001). Genetic engineering approaches to improve the bioavailability and the level of iron in rice grains. Theor. Appl. Genet..

[30] Ponstein A.S., Bade J.B., Verwoerd T.C., Molendijk L., Storms J., Beudeker R.F., Pen J. (2002). Stable expression of phytase (*phyA*) in canola (*Brassica napus*) seeds: towards a commercial product. Mol. Breed..

[31] Ullah A.H.J., Sethumadhavan K., Mullaney E.J., Ziegelhoffer T., Austin-Phillips S. (2002). Cloned and expressed fungal *phyA* gene in alfalfa produces a stable phytase. Biochem. Biophys. Res. Commun..

[32] Ullah A.H.J., Sethumadhavan K., Mullaney E.J., Ziegelhoffer T., Austin-Phillips S. (2003). Fungal *phyA* gene expressed in potato leaves produces active and stable phytase. Biochem. Biophys. Res. Commun..

[33] Xiao K., Katagi H., Harrison M., Wang Z.-Y. (2006). Improved phosphorus acquisition and biomass production in *Arabidopsis* by transgenic expression of a purple acid phosphatase gene from *M. truncatula*. Plant Sci..

[34] Pasamontes L., Haiker M., Henriquez-Huecas M., Mitchell D.B., van Loon A.P.G.M. (1997). Cloning of the phytases from *Emericella nidulans* and the thermophilic fungus *Talaromyces thermophilus*. Biochim. Biophys. Acta.

[35] Wyss M., Pasamontes L., Friedlein A., Remy R., Tessier M., Kronenberger A., Middendorf A., Lehmann M., Schnoebelen L., Röthlisberger U. (1999). Biophysical characterization of fungal phytases (myo-inositol hexakisphosphate phosphohydrolases): Molecular size, glycosylation pattern, and engineering of proteolytic resistance. Appl. Environ. Microbiol..

[36] Kniskern P.J., Hagopian A., Burke P., Schultz L.D., Montgomery D.L., Hurni W.M., Ip C.Y., Schulman C.A., Maigetter R.Z., Wampler D.E. (1994). Characterization and evaluation of a recombinant hepatitis B vaccine expressed in yeast defective for N-linked hyperglycosylation. Vaccine.

[37] Phillippy B.Q., Mullaney E.J. (1997). Expression of an *Aspergillus niger* phytase (*phyA*) in *Escherichia coli*. J. Agric. Food Chem..

[38] Kim S.-Y., Sohn J.-H., Pyun Y.-R., Choi E.-S. (2007). Variations in protein glycosylation in *Hansenula polymorpha* depending on cell culture stage. J. Microbiol. Biotechnol..

[39] Lee J., Park J.-S., Moon J.-Y., Kim K.-Y, Moon H.-M. (2003). The influence of glycosylation on secretion, stability, and immunogenicity of recombinant HBV pre-S antigen synthesized in *Saccharomyces cerevisiae*. Biochem. Biophys. Res. Commun..

[40] Lam J.S., Huang H., Levitz S.M. (2007). Effect of differential N-linked and O-linked mannosylation on recognition of fungal antigens by dendritic cells. PLoS One.

[41] Dasgupta S., Navarrete A.M., Bayry J., Delignat S., Wootla B., Andre S., Christophe O., Nascimbeni M., Jacquemin M., Martinez-Pomares L. (2007). A role for exposed mannosylations in presentation of human therapeutic self-proteins to CD4+ T lymphocytes. Proc. Natl. Acad. Sci. USA.

[42] Li H., D’Anjou M. (2009). Pharmacological significance of glycosylation in therapeutic proteins. Curr. Opin. Biotechnol..

[43] Schmidt F.R. (2004). Recombinant expression systems in the pharmaceutical industry. Appl. Microbiol. Biotechnol..

[44] Demain A.L., Vaishnav P. (2009). Production of recombinant proteins by microbes and higher organisms. Biotechnol. Adv..

[45] Walsh G. (2003). Biopharmaceutical benchmarks. Nat. Biotechnol..

[46] Bosch D., Castilho A., Loos A., Schots A., Steinkellner H. (2013). *N*-glycosylation of plant-produced recombinant proteins. Curr. Pharm. Des..

[47] Casey A., Walsh G. (2004). Identification and characterization of a phytase of potential commercial interest. J. Biotechnol..

[48] Protein blast. Blastp Program. http://blast.ncbi.nlm.nih.gov/Blast.cgi.

[49] Compute pI/*M*_W_ Tool. http://web.expasy.org/computre_pi.

[50] Cho J.-H., Yun S.-S., Jang Y.-K., Cha M.-J., Kwon N.-J., Chae S.-K. (2003). Identification and cloning of *jipA* enoding polypeptide that interacts with a homolog of yeast Rad6, UVSJ in *Aspergillus nidulans*. J. Microbiol..

[51] Laemmli U.K. (1970). Cleavage of structural proteins during the assembly of the head of bacteriophage T4. Nature.

[52] Pfaffl M.W., Dorak T. (2006). Relative quantification. Real-Time PCR.

[53] Dubray G., Bezard G. (1982). A highly sensitive periodic acid-silver stain for 1,2-diol groups of glycoproteins and polysaccharides in polyacrylamide gels. Anal. Biochem..

[54] Holman W.I. (1943). A new technique for the determination of phosphorus by the molybdenum blue method. Biochem. J..

[55] Murphy J., Riley J.P. (1962). A modified single solution method for the determination of phosphate in natural waters. Anal. Chim. Acta.

[56] Sims J.T., Pierzynski G.M. (2000). Soil test Phosphorus: Mehlich 3. Methods of Phosphorus Analysis for Soils, Sediments, Residuals, and Waters.

