# Supplementary material for: Expression of Aspergillus nidulans phy Gene in Nicotiana benthamiana Produces Active Phytase with Broad Specificities
Source: Int J Mol Sci. 2014 Sep 3;15(9):15571–91. doi: 10.3390/ijms150915571 (PMC4200759; doi:10.3390/ijms150915571)

Supplementary Information

Table S1. Daily body weight change of SD rats after oral administration of rPhy-P for 7 days.

	Dose (U/ kg)			
	0	500	1000	1500
Day 0	167.33 ± 3.055 [a]	154.67 ± 18.148 [a]	166.00 ± 5.292 [a]	153.33 ± 17.243 [a]
Day 1	171.33 ± 2.517 [b]	157.33 ± 17.673 [b]	171.00 ± 5.568 [b]	161.67 ± 17.559 [b]
Day 2	177.33 ± 1.155 [c]	162.67 ± 16.653 [c]	180.00 ± 7.211 [c]	166.67 ± 17.088 [c]
Day 3	179.33 ± 2.517 [d]	166.00 ± 18.028 [d]	181.67 ± 9.238 [d]	170.33 ± 18.230 [d]
Day 4	186.33 ± 5.508 [e]	171.33 ± 19.425 [e]	186.00 ± 8.718 [e]	178.67 ± 18.903 [e]
Day 5	180.67 ± 5.774 [f]	167.33 ± 19.425 [f]	180.00 ± 6.000 [f]	170.67 ± 20.817 [f]
Day 6	178.67 ± 7.572 [g]	166.67 ± 18.037 [g]	178.00 ± 8.718 [g]	169.33 ± 16.653 [g]
Day 7	180.67 ± 2.517 [h]	169.67 ± 18.230 [h]	183.33 ± 8.145 [h]	172.33 ± 15.948 [h]

No significance observed between normal and treated in any days. [a–h] showing no significant difference in days 0, 1, 2, 3, 4, 5, 6, 7, respectively.

Table S2. Organ weights of rats after oral administration of rPhy-P for 7 days.

Organs		Dose (U/ kg)			
		0 U	500 U	1000 U	1500 U
Heart		0.34 ± 0.014	0.37 ± 0.269	0.31 ± 0.386	0.35 ± 0.016
Liver		3.47 ± 0.164	3.36 ± 0.155	3.35 ± 0.540	3.57 ± 0.563
Spleen		0.24 ± 0.028	0.25 ± 0.017	0.25 ± 0.022	0.27 ± 0.051
Brain		1.00 ± 0.056	1.04 ± 0.138	1.03 ± 0.113	1.04 ± 0.172
Lung		0.49 ± 0.032	0.50 ± 0.033	0.53 ± 0.017	0.52 ± 0.060
Kidney	Left	0.39 ± 0.003	0.36 ± 0.037	0.38 ± 0.018	0.40 ± 0.010
	right	0.37 ± 0.005	0.38 ± 0.054	0.39 ± 0.014	0.41 ± 0.034

Table S3. Incidence of mortality and clinical signs after 7 days oral administration of rPhy-P into rats.

Dose (U/kg)	Mortality	Clinical Signs						
		Salivation	PRONE position	Decrease of Locomotive Activity	Loss of Fur	Bite/Scratch Wounds	Lacrimation	Closed Eyes
0	0/3 [1]	0/3	0/3	0/3	0/3	0/3	0/3	0/3
500	0/3	0/3	0/3	0/3	0/3	0/3	0/3	0/3
1000	0/3	0/3	0/3	0/3	0/3	0/3	0/3	0/3
1500	0/3	0/3	0/3	0/3	0/3	0/3	0/3	0/3

[1] Total number of rats treated.

Figure S1. Strategy for the site-directed mutagenesis (SDM) of pRTL2 vector sequences to insert *Aspergillus nidulans phy* gene. (**a**) Gene expression cassette in the pRTL2 vector contains 35S promoter, TEV leader sequence, *Nco*I site for the cloning and terminator. AT nucleotides in *Nco*I site were modified into GC nucleotides by SDM-PCR, changing to *Sac*II site; (**b**) *Aspergillus nidulans phy* gene was cloned into mutagenized pRTL2-MS vector, resulting in pRTL2-MS-Phytase. The resultant vector was used as a template to perform the secondary SDM-PCR to recover the start codon of *phy* gene, changing from *Sac*II to *Nco*I site; (**c**) *Phy* gene contains three *Nco*I sites.

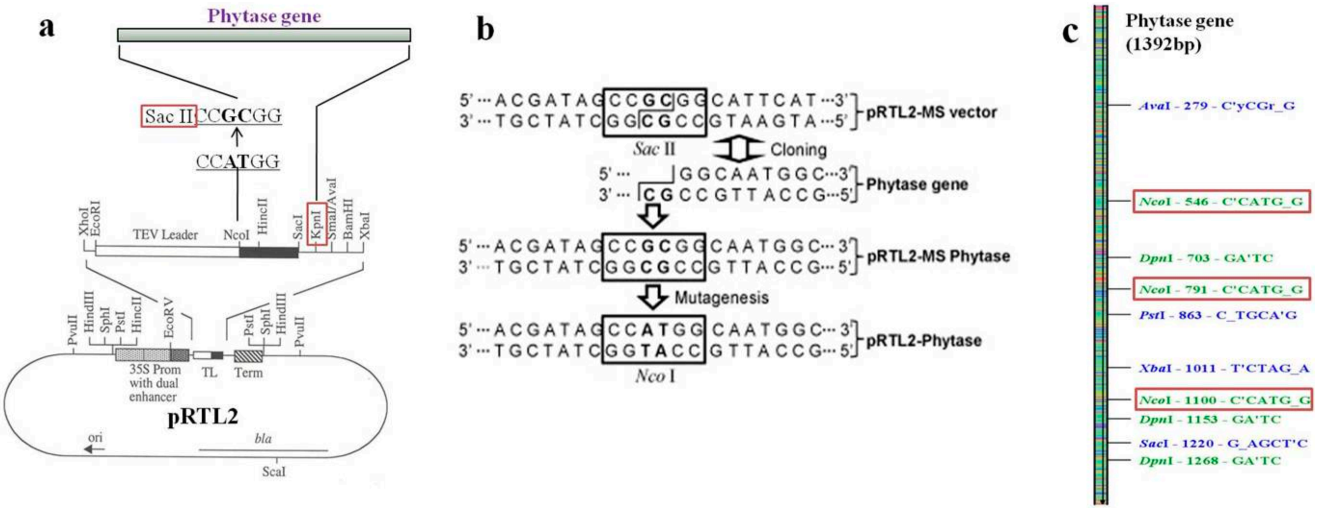

Supplement: Supplementary File 1 [file ijms-15-15571-s001.pdf]
